# A Rare Case Report With Literature Review of a Symptomatic Medial Discoid Meniscus in a 34-Year-Old Male: Arthroscopic Saucerization and Repair

**DOI:** 10.7759/cureus.102431

**Published:** 2026-01-27

**Authors:** Miguel Angel Palacios-Flores, Rolando O Suarez Peña, Margarita Chonate Correa

**Affiliations:** 1 Orthopaedics, Unidad de Rodilla - DOyT, EsSalud - Hospital Nacional Edgardo Rebagliati Martins, Lima, PER

**Keywords:** arthroscopic repair, congenita, medial discoid meniscus, nuclear magnetic resonance, saucerization

## Abstract

Medial discoid meniscus is a rare anomaly, with an incidence of 0.12-0.3%, predominantly in men under 18 years of age. This condition is characterized by a disk-shaped meniscus that covers a larger area of the tibial plateau, with increased thickness. It may be asymptomatic, and its diagnosis is based on nuclear magnetic resonance (MRI). Surgical treatment is reserved for symptomatic patients. Saucerization and suturing help preserve stability and as much meniscal tissue as possible; alternatively, a meniscectomy can be performed. The clinical case presented involves a 34-year-old man with chronic pain in the right knee and MRI findings indicating a medial discoid meniscus with a longitudinal and horizontal tear. Arthroscopy confirmed these findings, and saucerization and meniscal suturing were performed. After an uneventful recovery, the patient returned to normal activities in four months. This case shows an uncommon anomaly of the medial meniscus in an adult, characterized by its instability, highlighting the importance of evaluating its bilaterality and the injury pattern presented, allowing us to treat it appropriately.

## Introduction

The meniscus plays a crucial role in knee function by providing stability, reducing pressure on the articular cartilage, and expanding the contact area [1]. Variations in meniscal morphology more commonly affect the lateral meniscus than the medial; among these, the discoid meniscus (DM) stands out [2]. Key features distinguishing DM include its disk-like shape, increased thickness, and expanded coverage of the tibial plateau [3].

Medial discoid meniscus (MDM) is a rare anomaly, with an incidence ranging from 0.12% to 0.3% among patients presenting with knee pain and mechanical symptoms [4]. PubMed literature reports only about 80 documented cases [5]. Its etiology remains unclear, though hypotheses suggest developmental failures, such as incomplete separation between the medial meniscus and the anterior cruciate ligament (ACL) during weeks nine to 10 of gestation, potentially linked to genetic or familial factors [6]. MDM predominantly affects males, with 65% of cases occurring in individuals under 18 years of age.

## Case presentation

Medical history and clinical description

A 34-year-old male patient was referred to our hospital with a two-year history of right knee pain, unrelated to any apparent trauma. He reported no significant past medical history but mentioned undergoing arthroscopy for similar symptoms in the left knee 16 years prior (no records or images available). Pain intensity was rated 5/10 on the visual analog scale (VAS), exacerbated by flexion, and limiting his ability to jog or walk for extended periods.

Physical examination and diagnosis

**Figure 1:**
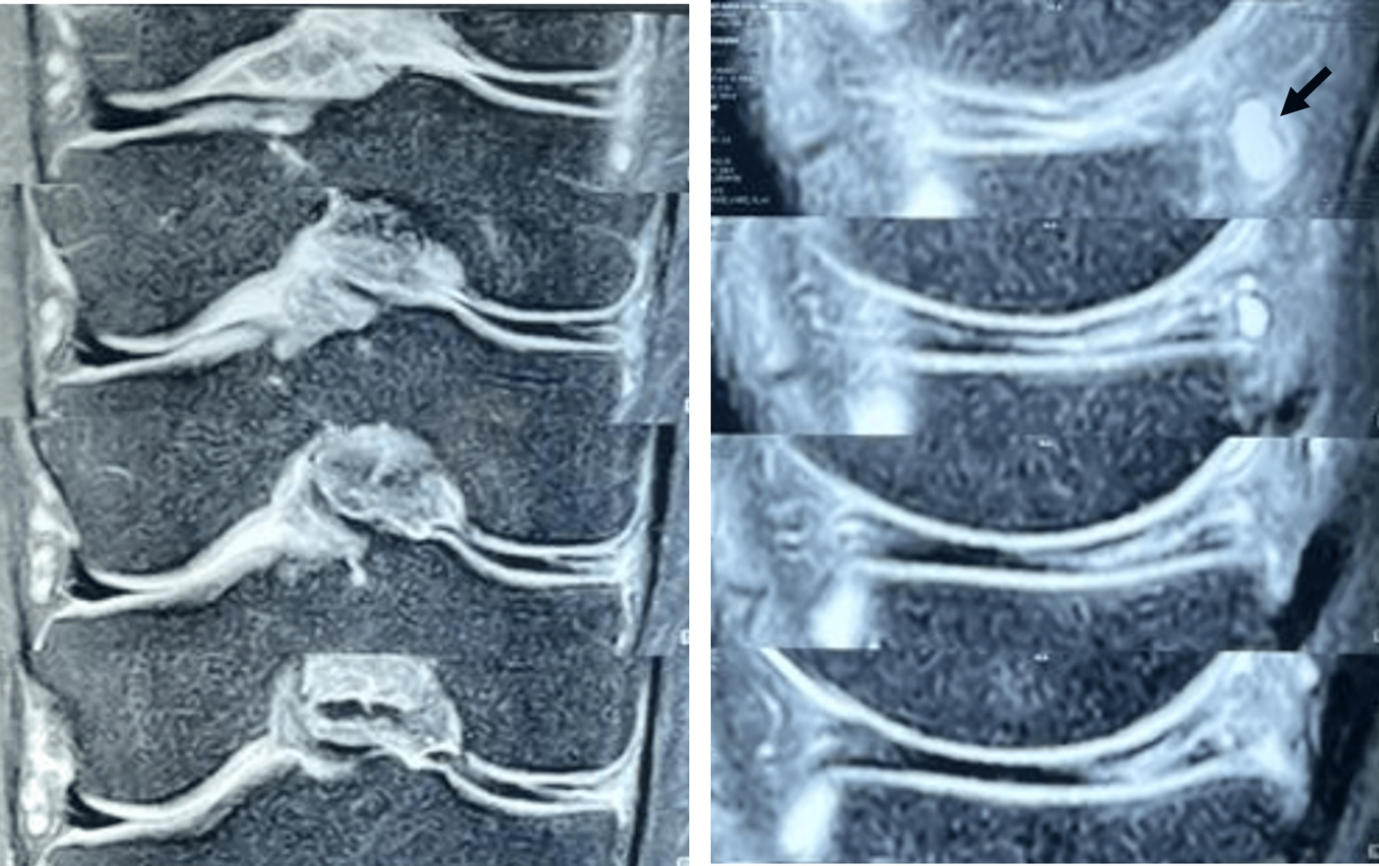
T2-weighted MRI showing the medial discoid meniscus in coronal (left) and sagittal (right) views, with the characteristic "bow-tie" sign evident in the sagittal view. The meniscal cyst is highlighted (arrow).

Physical examination revealed tenderness along the medial joint line and a positive McMurray test on the medial aspect of the right knee. Quadriceps muscle atrophy was noted in the right thigh, with strength graded 4/5 on the Daniels scale. No ligamentous instability, anterior knee pain, or malalignment was evident. Magnetic resonance imaging (MRI) of the right knee demonstrated a complete discoid configuration of the medial meniscus with a grade 3 vertical longitudinal tear extending from the body to the posterior horn, associated meniscal cysts, and scarring from a prior ACL injury (Figure 1).

Additional examination

Preoperative laboratory evaluations, including hematological, biochemical, and coagulation profiles, were unremarkable. Given the symptomatic discoid meniscal tear, arthroscopic intervention was recommended, with plans for potential saucerization and suture repair.

Intervention

**Figure 2:**
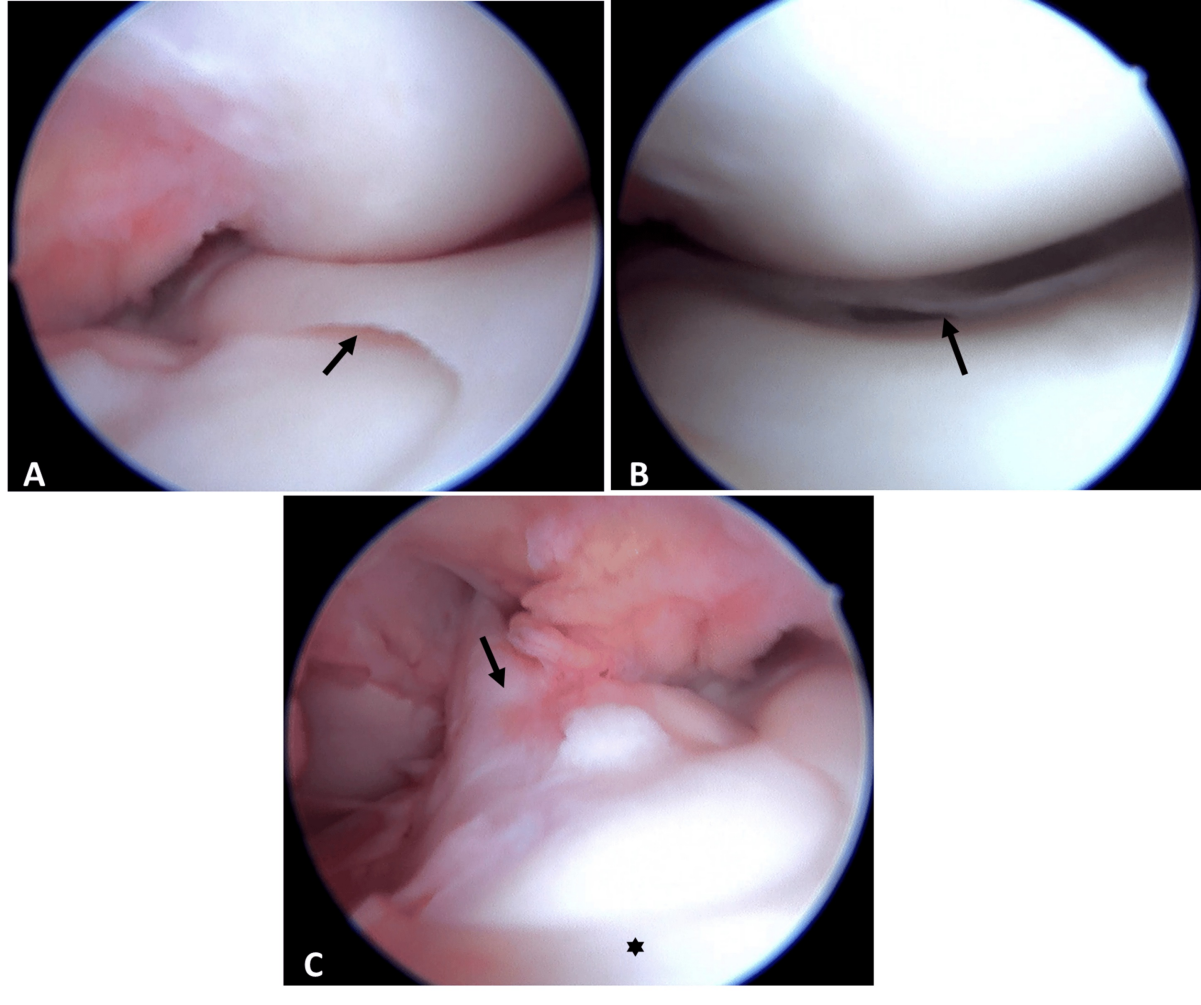
Arthroscopic views: (A) Confirmation of the medial discoid meniscus diagnosis based on the extent of medial meniscus coverage of the tibial plateau (arrow). (B) Longitudinal tear in the posterior horn (arrow). (C) Relationship between the anterior cruciate ligament (arrow) and the medial discoid meniscus (asterisk).

**Figure 3:**
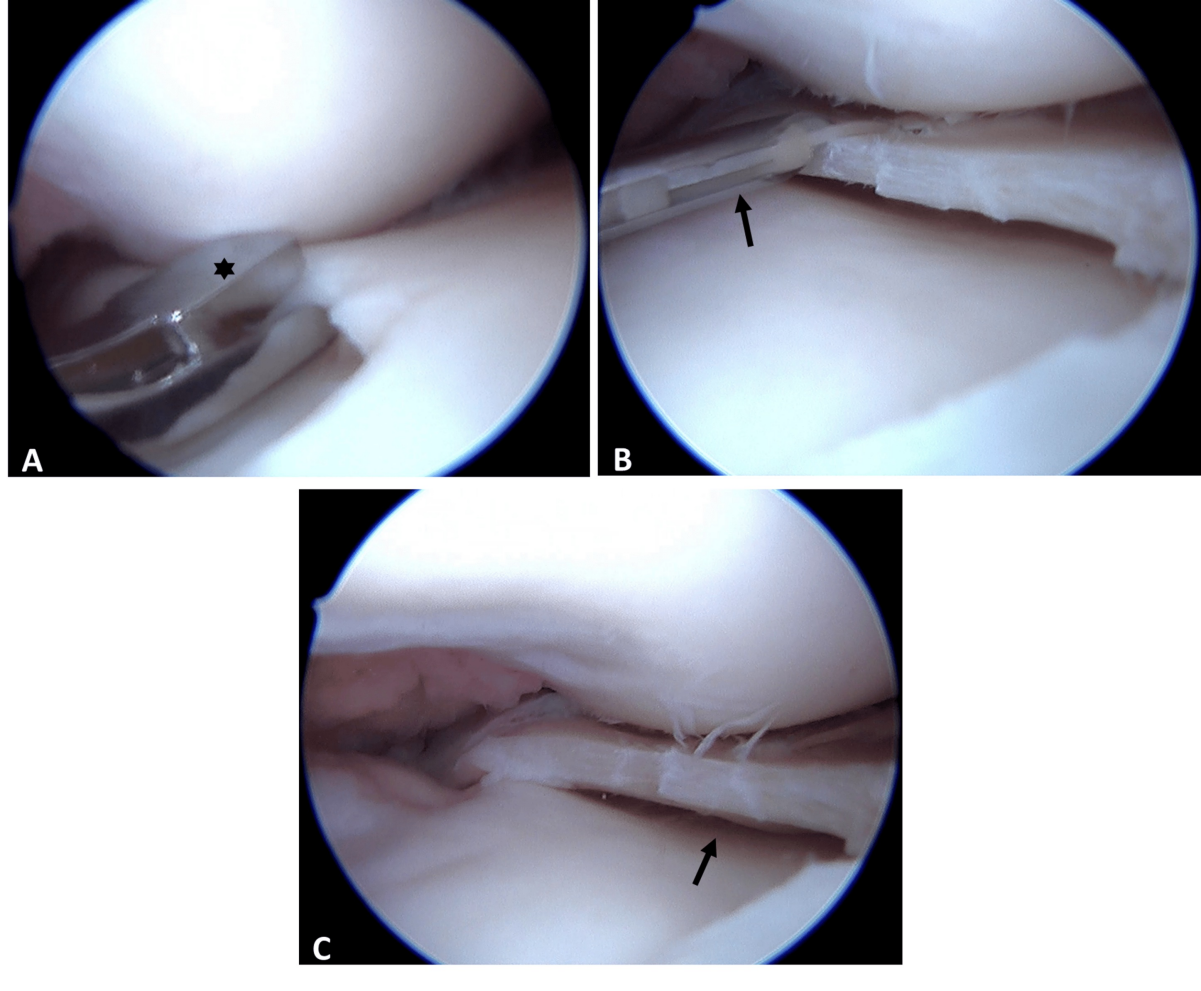
Arthroscopic views: (A) Saucerization of the medial discoid meniscus with a basket punch (asterisk). (B) Meniscal repair using all-inside vertical sutures (arrow) after saucerization. (C) Final result showing stable meniscal remnant (arrow).

Arthroscopy confirmed the MRI findings (Figure 2A), revealing a combined vertical longitudinal tear in the red-white zone involving the posterior horn (Figure 2B) and a horizontal tear affecting the capsular-attached meniscal remnant. Other structures, including ligaments, cartilage (Figure 2C), and the lateral meniscus, were intact. Based on these observations, saucerization was performed (Figure 3A), followed by meniscal repair using two all-inside vertical mattress sutures (Figures 3B-3C).

Outcome

The procedure was conducted on an outpatient basis, with the patient discharged uneventfully after initial recovery. Physiotherapy commenced one week after posterior skin suture removal, with non-weight-bearing for the first four weeks. After the four-week follow-up, partial weight-bearing with crutches was initiated. By two months, unassisted ambulation was permitted, and full physiotherapy completion allowed unrestricted return to activities by four months.

## Discussion

MDM is a rare anomaly first described in 1930 by Watson-Jones in a 34-year-old patient during an open meniscal procedure [7]. Its true incidence may be underestimated due to asymptomatic cases or underreporting [8]. Most documented instances occur in pediatric or adolescent males under 18 years [9], with horizontal tears being the predominant lesion type [6]. In contrast, our patient presented symptoms at 34 years of age, featuring a combined vertical longitudinal and horizontal tear in the meniscal remnant.

The etiology of MDM remains elusive. One theory posits a failure in central meniscal involution during fetal development, as proposed by Smillie [10], while another suggests an acquired biomechanical mechanism leading to hypermobility, according to Kaplan [11]. Macroscopically, MDM exhibits altered thickness and shape. Histologically, it displays disorganized circumferential collagen fibers, reduced vascularity, and deficient capsular attachments, rendering it more vulnerable to injury from rotational forces in the medial compartment [6]. Occasionally, the anterior horn may fuse with the tibial insertion of the ACL [12]. In our case, while meniscal thickness was notably altered (Figure 3C), no such fusion with the ACL was observed (Figure 2C).

Clinically, MDM manifests symptoms like typical meniscal tears, including instability, joint effusion, locking, limping, or audible snapping, often with a positive medial McMurray test and effusion [11]. Our patient exhibited medial joint line pain and a positive McMurray sign, aligning with these descriptions. Smillie's embryological classification was among the earliest for these anomalies [10]. In 1962, Watanabe pioneered the arthroscopic evaluation of DM and introduced a classification system dividing them into complete, incomplete, and Wrisberg-ligament variants [13]. Our case was classified as a complete MDM, as confirmed arthroscopically (Figure 2A).

Diagnosis relies primarily on the knee MRI. Araki et al. established a coronal-view cutoff of 14 mm for meniscal width on the tibial plateau, yielding 93.2% diagnostic accuracy [14]. Samoto et al. proposed criteria including a meniscal radius (>20% in coronal section: minimum meniscal width/maximum tibial diameter) and meniscal coverage (>75% in sagittal section: sum of anterior and posterior horns/maximum meniscal diameter), achieving 95% sensitivity and 97% specificity when combined [15]. We applied Samoto's criteria, measuring a 34% meniscal radius and 84% coverage, confirming the MDM diagnosis.

Treatment for MDM is similar to that of lateral DM [16]. Asymptomatic cases warrant conservative management, with surgery reserved for symptomatic or torn lesions [17]. Saucerization is the preferred approach, often augmented by suturing for remnant instability, as evidenced in Anderson et al.’s pediatric series, where seven of 22 medial discoid menisci required repair [18]. Preservation via repair is advisable to mitigate long-term degenerative risks associated with resection [9]. In our patient, saucerization (Figure 3A) combined with meniscal suturing (Figure 3B) minimized tissue loss, restored stability, and promoted healing, following the techniques described by Sevillano-Pérez et al [19].

At the six-month follow-up, the patient reported no complications and expressed satisfaction, having resumed normal activities. This represents the first documented MDM case at our institution, precluding comparative series analysis. The absence of records from the contralateral knee arthroscopy 16 years prior hinders confirmation of potential bilaterality.

## Conclusions

This case report presents a rare symptomatic MDM-the first at our institution-in a 34-year-old male with adult-onset symptoms and a unique vertical longitudinal plus horizontal tear, without ACL fusion. MRI diagnosis using established criteria (in our case: 34% radius and 84% meniscal coverage) guided arthroscopic saucerization and vertical suturing, resulting in full functional recovery within four months. This case report highlights bilaterality evaluation and conservative tissue preservation to prevent degeneration, and contributes insights to the limited adult MDM literature, urging thorough assessment in chronic non-traumatic knee pain.
